# The presentation of depression symptoms in attention‐deficit/hyperactivity disorder: comparing child and parent reports

**DOI:** 10.1111/camh.12253

**Published:** 2017-11-10

**Authors:** Annie Fraser, Miriam Cooper, Sharifah Shameem Agha, Stephan Collishaw, Frances Rice, Anita Thapar, Olga Eyre

**Affiliations:** ^1^ MRC Centre for Neuropsychiatric Genetics and Genomics School of Medicine Cardiff University Wales UK; ^2^ Child and Adolescent Mental Health Services Network (CAMHS) Cwm Taf University Health Board UK

**Keywords:** Attention‐deficit/hyperactivity disorder, depression, symptomatology

## Abstract

**Background:**

Attention‐deficit/hyperactivity disorder (ADHD) frequently co‐occurs with depression, and outcomes are poor when both are present. Little is known about whether depression symptoms present differently in ADHD compared to the general population, or how reliable young people with ADHD are at reporting these symptoms. This study aimed to describe depression symptoms in a clinical ADHD sample compared to a population sample, and compare self‐reports of depression symptoms with parent‐reports.

**Methods:**

Two hundred and forty‐nine children with ADHD and their parents completed follow‐up questionnaires around 5 years after taking part in a Cardiff University ADHD study. Child depression symptoms were measured using parent‐ and child‐reported Mood and Feelings Questionnaires (MFQ) and compared to a population sample with MFQ data (*n* = 1460). Within both samples, child‐ and parent‐reported depression symptoms were compared.

**Results:**

Although the profile of depression symptoms was similar between young people with ADHD and those in the general population, depression symptoms were much more common in the ADHD sample (parent‐rated MFQ score = 24.52 vs. 9.39; child‐rated = 21.02 vs. 11.86). The most common symptoms in both samples included irritability, restlessness and concentration difficulties, with core depression symptoms such as feeling miserable/unhappy also prominent. Within the ADHD sample, but not the population sample, children reported depression symptoms less frequently than their parents.

**Conclusions:**

Young people with ADHD are at high risk of experiencing symptoms of depression but may under‐report the severity of their symptoms. Obtaining parent reports of depression symptoms in this group may be important to avoid missing key indicators of risk.

**Key Practitioner Message**

- Depression symptoms, as measured by the Mood and Feelings Questionnaire, are common in children and adolescents with ADHD.
- The profile of depression symptoms in ADHD is similar to that in the general population.
- Children with ADHD report lower levels of depression symptoms than their parents, the opposite pattern to the general population.
- Children with ADHD may under‐report the severity of their own symptoms of depression.

## Introduction

Attention‐deficit/hyperactivity disorder (ADHD) is a neurodevelopmental disorder characterised by hyperactive‐impulsiveness and inattention (APA, 2013). In addition to these core symptoms, ADHD often co‐occurs with other neurodevelopmental conditions, behavioural disorders and emotional disorders (Thapar, Cooper, & Rutter, 2016), with over half thought to have one or more comorbid disorders (Acosta et al., 2008). Emotional disorders in children and adolescents with ADHD can be hard to identify as they have a less overt presentation than behavioural difficulties and are often considered less problematic by parents and teachers (Tandon, Cardeli, & Luby, 2009). Depression, in particular, occurs in children and adolescents with ADHD at a higher rate than in those without ADHD (Daviss, 2008), with a recent literature review (Meinzer, Pettit, & Viswesvaran, 2014) finding rates of unipolar depression in ADHD to be consistently elevated across studies.

It is important to recognise children with ADHD who present with symptoms of depression, as its presence alongside ADHD is associated with poorer academic and social functioning (Becker, Langberg, Evans, Girio‐Herrera, & Vaughn, 2015; Blackman, Ostrander, & Herman, 2005). Additionally, depression itself is more severe when comorbid with ADHD, with evidence suggesting an earlier onset, a longer duration, greater depression‐associated impairment, a higher suicidality rate and a greater likelihood of psychiatric hospitalisation (Biederman et al., 2008). Evidence also suggests that depression symptoms may mediate the increased risk for suicide seen in ADHD (Balazs, Miklosi, Kereszteny, Dallos, & Gadoros, 2014; James, Lai, & Dahl, 2004).

With this in mind, early identification of depression is crucial. However, in children and adolescents with ADHD, its identification can be complicated. Firstly, depression has multiple symptoms which overlap both with ADHD core symptoms, and with other non‐core symptoms that commonly occur with ADHD (Daviss, 2008). Examples include restlessness and concentration difficulties (which are core ADHD symptoms), and irritability that commonly co‐occurs with ADHD (Shaw, Stringaris, Nigg, & Liebenluft, 2014). Secondly, medication used to treat ADHD can have side effects such as insomnia and weight loss (Daviss, 2008), both of which are features of depression. Therefore it is useful to understand how depression presents in ADHD as there may be symptom differences that distinguish it from depression in those without ADHD. Research has found that depressive cognitions, social withdrawal, suicidal thoughts, psychomotor retardation and anhedonia are better discriminators of depression in ADHD than symptoms such as restlessness, indecisiveness, and an inability to concentrate (Diler et al., 2007). However, there are few studies that explore how depression presents in ADHD when compared to the general population.

Furthermore, when examining psychopathology in children and adolescents, especially in the context of ADHD, it is worth considering the informant. Studies consistently find parent‐child agreement to be low to moderate when measuring psychopathology (Rey, Schrader, & Morris‐Yates, 1992; Van der Meer, Dixon, & Rose, 2008), particularly for internalising psychopathology. The reason for this low agreement is complex. Younger children are thought to be less reliable at reporting their own symptoms than adolescents due to limitations in their understanding, but adolescents are more reliable than their parents for reasons which include reduced parental involvement in their day‐to‐day lives (Klein, Dougherty, & Olino, 2005). Furthermore, informant reliability is also thought to vary depending on various factors such as family functioning, economic and social situation, maternal depression and the health condition of the child (Collishaw, Goodman, Ford, Rabe‐Hesketh, & Pickles, 2009). Overall, both parent and child informants are important to gain an adequate clinical picture for most psychopathologies (Jensen et al., 1999), but self‐reports are especially important for depression symptoms in older children and adolescents. This is important to consider in an ADHD sample, as previous research has found that individuals with ADHD under‐report their own ADHD symptoms relative to other informants (Danckaerts, Heptinstall, Chadwick, & Taylor, 1999), which may suggest that self‐report of psychopathology in ADHD is poor. At present, little is known about the agreement between parent and child report of depression symptoms in ADHD.

This study utilised a clinical sample of adolescents with ADHD and a general population sample to address the following aims: (a) compare the prevalence of depression symptoms, (b) compare the depression symptoms most commonly reported, and (c) examine differences between child‐ and parent‐reports of depression symptoms.

## Methods

### Clinical ADHD sample

This study used a subsample of children originally recruited as part of the Cardiff University Study of ADHD Genes and Environment (SAGE). The SAGE study consisted of 696 children and adolescents recruited between 2007 and 2011 from child psychiatry and paediatric clinics across South Wales. The children of this original sample were aged 6–18 years (mean = 10.9 years), 84% were male, and all were of British Caucasian origin. All had a clinical and research diagnosis of ADHD. To examine the prevalence of depression symptoms in this sample in adolescence, follow up postal questionnaires (including the Mood and Feelings Questionnaire) were sent out to a subsample of 434 participants in 2014–2015. The subsample was selected on the basis that children were aged 12 years or under at the time of the initial data collection (mean = 9.0 years) and that the family had consented to be contacted for future research at the time of the original study. Based on data available from a structured clinical interview at follow up (the Development and Well Being Assessment (DAWBA), (Goodman, Ford, Richards, Gatward, & Meltzer, 2000)), 66% of adolescents in this subsample still have a probable ADHD diagnosis, the majority of the remaining 34% still report the presence of ADHD symptoms, and just over half the subsample are still being treated with ADHD medication. This is in line with previous rates reported from another study, where the majority of participants continue to have ADHD (Langley et al., 2010).

### Population sample

The general population sample came from the Cardiff Study of All Wales and North West of England Twin register, which contains data from all twins who were born in Wales or the Greater Manchester area between 1980 and 1991. As part of the data collection, parents and twins older than 11 years were sent postal questionnaires (including the Mood and Feelings Questionnaire) for completion, from which there were *N* = 1463 families who responded (Rice, Harold, & Thapar, 2002). For this paper, only data from the eldest twin were used.

### Ethical considerations

Ethical approval was obtained for the SAGE study from the Wales Multicentre Research Ethics Committee (MREC). Written informed consent from parents and assent from children (or consent if aged 16 and older) were obtained for all individuals. The twin study was approved by Wales MREC and Northwest MREC and consent was obtained from all participants following completion of the questionnaire.

### Measures

Parents and children completed the Mood and Feelings Questionnaire (MFQ) (Angold et al., 1995) which is a widely used depression screening instrument. It includes 34‐items with each item scored from 0 to 2, with 0 being ‘not true’, 1 being ‘sometimes true’ and 2 being ‘true’ giving a range of scores from 0 to 68. Widely accepted optimum cut‐offs for the MFQ are ≥27 for the child‐report and ≥21 for the parent‐report (Wood, Kroll, Moore, & Harrington, 1995).

### Analyses

Total parent‐ and child‐reported MFQ scores were calculated for each sample and compared using independent sample *t*‐tests. Where >10% of the MFQ items (>3) were missing for an individual, the total score was left as missing. Where <10% (≤3) of MFQ items were missing, a mean score of the completed items was imputed allowing a total to be calculated.

In order to identify the most frequently endorsed items, a mean score was calculated for each MFQ item (both for parent and child measures). This allowed items to be ranked from the most commonly endorsed to the least commonly endorsed. The most commonly reported symptoms in the ADHD sample were then compared to the population sample. In order to assess agreement between parent and child reports in each of the samples, the Pearson's correlation between total parent and child MFQ score was calculated. In addition, the kappa coefficient of agreement was calculated for each individual MFQ item (‘true’ and ‘sometimes’ vs. ‘not true’).

As the ADHD sample was older than the population sample (mean 14.6 vs. 12.8 years), analyses were repeated excluding the oldest participants from the ADHD sample (age ≥16 years) making the mean ages more comparable (13.1 vs. 12.8 years). As there were prominent gender differences between the two samples, analyses were also repeated for boys and girls separately.

Data were analysed using SPSS version 20.

## Results

### Sample demographics and response

In the ADHD sample, the mean age was 14.6 years (range 8–20 years) and included 81.9% males and 18.1% females. In the population sample, the mean age was 12.8 years (range 8–17 years) (Van Den Bree et al., 2007), and included 46.1% males and 53.9% females.

**Figure 1:**
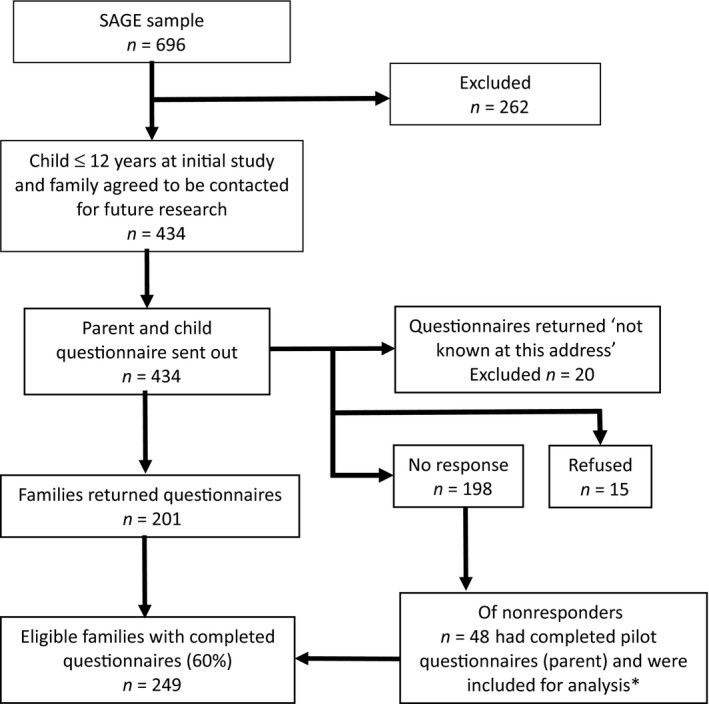
Flow chart of participants for Study of ADHD and Mood. SAGE, Study of ADHD, Genes and Environment. *Pilot MFQ data were collected in 2013–2014. Although the children in the pilot questionnaire group were younger than the rest of the sample, the MFQ scores did not significantly differ so they were included in the analysis

MFQ data were available for 249 families in the clinical ADHD sample, for whom 171 had both parent‐ and child‐report data. Two families were excluded from the analysis, due to >10% of MFQ items missing from either the parent or child report, leaving a total of 247 families, *n* = 167 who had both parent‐ and child‐report data. Details of the study process are shown in Figure 1.

Those returning questionnaires were slightly younger and less likely to be from low income families than those who did not return them. However, there were no differences in child or parent psychopathology at study baseline between responders and non‐responders. The mean total MFQ score was higher in girls than in boys with a mean of 28.2 (*SD* = 15.5) versus 23.5 (*SD* = 15.2) on the parent‐rated measure (*t*(244) = 1.873, *p* = .066) and 25.0 (*SD* = 15.0) versus 20.2 (*SD* = 15.4) on the child‐rated measure (*t*(168) = 1.51, *p* = .132), but MFQ score was not associated with age.

In the population sample 1460 families had MFQ data available for twin 1 (1456 parent and 913 child), 909 of whom had both parent‐ and child‐report data. Nine families were excluded from the analysis due to >10% of MFQ items missing from either the parent or child report, leaving a total of 1451 families, *n* = 901 who had both parent‐ and child‐report data. The mean total MFQ score was higher in girls than in boys with a mean of 9.9 (*SD* = 9.24) versus 8.8 (*SD* = 8.01) on the parent rated measure (*t*(1437) = 2.38, *p* = .017) and 12.9 (*SD* = 10.54) versus 10.6 (*SD* = 9.61) on the child rated measure (*t*(905) = 3.38, *p* = .001).

### Prevalence of depression symptoms

As shown in Table 1, the mean totals for both the parent‐report MFQ (pMFQ) and child‐report MFQ (cMFQ) were significantly higher in the clinical ADHD sample than in the population sample (mean pMFQ score = 24.5 vs. 9.4, *t*(269.9) = 14.94, *p* < .001, mean cMFQ score = 21.0 vs. 11.9, *t*(197.5) = 7.46, *p* < .001). These results remained similar when the oldest children were removed from the ADHD sample (mean pMFQ score = 25.3 vs. 9.4, *t*(171) = 13.16, *p* < .001, mean cMFQ score = 20.4 vs. 11.9, *t*(117.8) = 6.0, *p* < .001) and when the sample was split by gender (in boys: mean pMFQ 23.5 vs. 8.8, *t*(231.6) = 13.2, *p* < .001; mean cMFQ 20.2 vs. 10.7, *t*(80.2) = 6.93, *p* < .001. In girls: mean pMFQ 28.2 vs. 9.9, *t*(45.8) = 7.84, *p* < .001; mean cMFQ 25.0 vs. 12.9, *t*(28.5) = 4.24, *p* < .001).

**Table 1:** Comparing MFQ scores in ADHD and population samples

	ADHD sample mean (*SD*)	Population sample mean (*SD*)	T statistic (*df*)	*p* value
pMFQ	24.52 (15.40)	9.39 (8.72)	14.94 (269.9)	<.001
cMFQ	21.02 (15.40)	11.86 (10.19)	7.46 (197.5)	<.001

MFQ, Mood and Feelings Questionnaire; pMFQ, parent‐rated MFQ; cMFQ, child‐rated MFQ; ADHD, Attention‐Deficit/Hyperactivity Disorder.

The proportion scoring above the clinical cut‐off was also much higher in the ADHD sample than in the population sample (parent‐report 54.5% vs. 10.6%, *χ* ^2^ = 285.24, *p* < .001; child‐report 32.4% vs. 10.5%, *χ* ^2^ = 56.73, *p* < .001).

Within the ADHD sample the pMFQ mean total was higher than the cMFQ mean total, whereas within the population sample the cMFQ mean total was higher than the pMFQ total. This pattern remained the same regardless of gender.

### Description of depression symptoms in clinical ADHD sample

The highest scoring symptom on parent‐report was feeling grumpy/irritable with parents followed by difficulty concentrating and restlessness. On the child‐reported measure the highest scoring symptom was difficulty concentrating followed by restlessness and feeling grumpy with parents. Suicidal thoughts and symptoms of psychomotor and cognitive retardation (i.e. talking more slowly than usual, moving and walking more slowly than usual, and sleeping more than usual) were the lowest scoring symptoms on both parent‐ and child‐report. The frequency at which each MFQ item is reported for pMFQ and cMFQ is detailed in Table 2.

**Table 2:** The frequency at which each MFQ item is endorsed in the ADHD sample

MFQ Item	Mean Item Score (range 0–2)	
	Parent‐rated	Child‐rated
1. S/he felt miserable or unhappy.	**1.17**	0.87
2. S/he didn't enjoy anything at all.	0.70	0.56
3. S/he was less hungry than usual.	0.71	0.59
4. S/he ate more than usual.	0.68	0.67
5. S/he felt so tired s/he just sat around and did nothing.	0.80	0.83
6. S/he was moving and walking more slowly than usual.	0.36	0.41
7. S/he was very restless.	**1.28**	**1.11**
8. S/he felt s/he was no good anymore.	0.82	0.55
9. S/he blamed him/herself for things that weren't his/her fault.	0.64	0.57
10. It was hard for him/her to make up his/her mind.	**1.13**	**1.07**
11. S/he felt grumpy and cross with his/her parents.	**1.49**	**1.08**
12. S/he felt like talking less than usual.	0.68	0.69
13. S/he was talking more slowly than usual.	0.24	0.31
14. S/he cried a lot.	0.47	0.40
15. S/he thought there was nothing good for him/her in the future.	0.73	0.64
16. S/he thought that life wasn't worth living.	0.47	0.46
17. S/he thought about death or dying.	0.43	0.42
18. S/he thought his/her family would be better off without him/her.	0.50	0.45
19. S/he thought about killing him/herself.	0.31	0.30
20. S/he didn't want to see his/her friends.	0.61	0.45
21. S/he found it hard to think properly or concentrate.	**1.41**	**1.16**
22. S/he thought bad things would happen to him/her.	0.51	0.45
23. S/he hated him/herself.	0.67	0.54
24. S/he felt s/he was a bad person.	0.54	0.53
25. S/he thought s/he looked ugly.	0.53	0.51
26. S/he worried about aches and pains.	0.77	0.54
27. S/he felt lonely.	0.75	0.58
28. S/he thought nobody really loved him/her.	0.65	0.47
29. S/he didn't have any fun at school.	0.88	0.69
30. S/he thought s/he could never be as good as other kids.	0.82	0.71
31. S/he felt s/he did everything wrong.	0.72	0.52
32. S/he didn't sleep as well as s/he usually sleeps.	1.00	**0.90**
33. S/he slept a lot more than usual.	0.35	0.35
34. S/he wasn't’ as happy as usual, even when s/he was praised or rewarded	0.69	0.58

ADHD, Attention‐Deficit/Hyperactivity Disorder. Mean scores were calculated for each MFQ item allowing them to be ranked from highest to lowest according to how common they were. The five most frequently endorsed items for both parent and child‐rated MFQ are highlighted in bold.

### Description of depression symptoms in population sample

For both parent‐ and child‐report, the highest scoring symptom was feeling grumpy/irritable with parents followed by feeling miserable or unhappy. The lowest scoring symptom for both was thoughts of killing self. The frequency at which each MFQ item is reported for pMFQ and cMFQ is detailed in Table 3.

**Table 3:** The frequency at which each MFQ item is endorsed in the population sample

MFQ Item	Mean Item Score (range 0–2)	
	Parent‐rated	Child‐rated
1. S/he felt miserable or unhappy.	**0.70**	**0.65**
2. S/he didn't enjoy anything at all.	0.25	0.17
3. S/he was less hungry than usual.	0.30	0.46
4. S/he ate more than usual.	**0.45**	0.49
5. S/he felt so tired s/he just sat around and did nothing.	0.42	**0.56**
6. S/he was moving and walking more slowly than usual.	0.15	0.26
7. S/he was very restless.	0.40	**0.50**
8. S/he felt s/he was no good anymore.	0.25	0.24
9. S/he blamed him/herself for things that weren't his/her fault.	0.24	0.28
10. It was hard for him/her to make up his/her mind.	**0.59**	**0.62**
11. S/he felt grumpy and cross with his/her parents.	**0.86**	**0.73**
12. S/he felt like talking less than usual.	0.37	0.41
13. S/he was talking more slowly than usual.	0.08	0.13
14. S/he cried a lot.	0.27	0.29
15. S/he thought there was nothing good for him/her in the future.	0.13	0.27
16. S/he thought that life wasn't worth living.	0.07	0.17
17. S/he thought about death or dying.	0.14	0.24
18. S/he thought his/her family would be better off without him/her.	0.08	0.18
19. S/he thought about killing him/herself.	0.03	0.09
20. S/he didn't want to see his/her friends.	0.16	0.19
21. S/he found it hard to think properly or concentrate.	0.42	**0.50**
22. S/he thought bad things would happen to him/her.	0.14	0.32
23. S/he hated him/herself.	0.15	0.29
24. S/he felt s/he was a bad person.	0.11	0.23
25. S/he thought s/he looked ugly.	0.25	**0.50**
26. S/he worried about aches and pains.	**0.43**	0.41
27. S/he felt lonely.	0.28	0.35
28. S/he thought nobody really loved him/her.	0.24	0.22
29. S/he didn't have any fun at school.	0.33	0.40
30. S/he thought s/he could never be as good as other kids.	0.28	0.33
31. S/he felt s/he did everything wrong.	0.20	0.22
32. S/he didn't sleep as well as s/he usually sleeps.	0.22	0.46
33. S/he slept a lot more than usual.	0.21	0.38
34. S/he wasn't as happy as usual, even when s/he was praised or rewarded	0.23	0.30

ADHD, Attention‐Deficit/Hyperactivity Disorder. Mean scores were calculated for each MFQ item allowing them to be ranked from highest to lowest according to how common they were. The five most frequently endorsed items for both parent and child‐rated MFQ are highlighted in bold.

### Comparison between parent‐report and child‐report in clinical ADHD sample

Pearson's correlation for total pMFQ and cMFQ in the ADHD sample was 0.642 (*p* < .001). When assessing agreement, for the majority of individual MFQ items, parents were more likely to score the symptom as present than the child. The items with the highest agreement included thoughts of killing self, not having fun at school and crying a lot. Items with the lowest agreement included restlessness, difficulty concentrating and feeling grumpy with parents. The kappa coefficients ranged from 0.634 to 0.100 (detailed results available on request).

### Comparison between parent‐report and child‐report in population sample

Pearson's correlation between total pMFQ and cMFQ score was 0.471 (*p* < .001). When assessing agreement, unlike for the ADHD group, the children were more likely to score symptoms as present than their parents. The items with the highest agreement included feeling miserable or unhappy, crying a lot and thoughts of looking ugly. Items with the lowest agreement included sleeping more than usual, talking more slowly than usual and moving and walking more slowly than usual. The Kappa coefficients ranged from .358 to .102.

## Discussion

### Prevalence and description of depression symptoms

The results suggest that depression symptoms are common in ADHD. The mean MFQ score in the ADHD sample was significantly higher than in the population sample on both child‐ and parent‐report measures. Both parent‐ and child‐rated MFQ scores in our sample were higher than previous studies have found in both a population sample (Sund, Larsson, & Wichstrom, 2001) and a non‐depressed ADHD sample (Diler et al., 2007). The findings are of clinical significance as the mean pMFQ score of 24.52 is well above the validated cut‐off of 21 for suspected depression. Over half of the ADHD sample fell into this high‐risk category.

Amongst the most common depression symptoms found in our ADHD sample were difficulty concentrating, restlessness and feeling grumpy with parents. These symptoms overlap with those of ADHD, so it is unsurprising that they were common in our sample. Previous research has found that these symptoms do not distinguish well between children with both ADHD and depression and those with ADHD alone (Diler et al., 2007). This could suggest that depression scores in this sample are artificially elevated by symptoms which overlap with ADHD symptoms. However, these symptoms are also the most frequently endorsed in our population sample, suggesting that it is not specific to ADHD, but a consistent finding when using the MFQ in this age group. This is in line with previous research in another general population sample (Crowe, Ward, Dunnachie, & Roberts, 2006). With this in mind, it may be important in ADHD to focus on a change or worsening in these symptoms as an indicator of depression, rather than looking at them at an absolute level. The MFQ asks for a 2‐week time‐frame of symptoms, so in theory this should be the case. However, children with ADHD can have a distorted perception of time (Barkley, 1997; Toplak & Tannock, 2005), so they may not reliably report to this timeframe.

It is also important to note that common side effects of ADHD medication include insomnia and weight loss. Therefore, these symptoms could elevate MFQ scores in the ADHD sample. However, as was the case for symptoms of restlessness and concentration difficulties, the frequency of insomnia and weight loss were not reported relatively more frequently in the ADHD sample than the population sample when compared to the other MFQ items. If anything, they were reported relatively less frequently in the ADHD sample.

Overall differences in the relative frequency of symptoms seen between the ADHD group and the population sample were minor. In both samples, feeling grumpy with parents, difficulty in making up their mind, feeling miserable or unhappy, difficulty concentrating and restlessness were amongst the most frequently reported symptoms. In the ADHD sample, symptoms such as appetite and sleep disturbance and psychomotor retardation were less frequently reported in comparison to the other MFQ items. In the population sample, crying a lot and thoughts of looking ugly were comparatively more common symptoms. However, the population sample contained a larger proportion of girls, and symptoms of sadness, guilt and worries about body image have been found to be more common in females than in males (Bennett, Ambrosini, Kudes, Metz, & Rabinovich, 2005). The symptoms ‘I thought I could never be as good as the other kids’, ‘I thought there was nothing good for me in the future’ and ‘I felt I was no good anymore’ were also common in the ADHD group. This is consistent with research showing that children and adolescents with ADHD have lower self‐esteem (Shaw‐Zirt, Popali‐Lehane, Chaplin, & Bergman, 2005; Slomkowski, Klein, & Mannuzza, 1995), particularly those with comorbid internalising disorders (Bussing, Zima, & Perwien, 2000). It is also important to note that although the depression symptoms reported as indicating more severe symptomatology (e.g. suicidality, weight/appetite and psychomotor disturbance) (Cole et al., 2011) were less commonly endorsed compared to other symptoms in the ADHD sample, they were still substantially more common than in the twin sample. In particular, symptoms of suicidality (‘I thought about killing myself’) were present in 20%–25% of the ADHD sample, according to both parent‐report and child‐report, compared to 2%–7% of the population sample. This identifies this ADHD sample as having not only a higher prevalence of depression symptoms, but also to be at greater risk of high severity depression than the population sample, and highlights the need for caution in generalising data from population samples to clinical samples.

### Comparison between parent‐report and child‐report

The six most frequently endorsed items on the MFQ in our ADHD sample were common to both parent‐ and child‐reports, suggesting that parents and children are identifying the same symptoms. However, there are findings emerging from this study that are important to address.

Firstly, in the ADHD sample, the cMFQ mean was lower than the pMFQ mean. This differs to our population sample, and to other research on the MFQ in non‐ADHD samples. It has been previously understood that when using depression screening tools, child‐report versions score higher than parent‐report versions, leading to the cut‐off point for depression being higher for the cMFQ (≥27) than for the pMFQ (≥21) (Wood et al., 1995). The fact that our results show the opposite could suggest that children with ADHD are either poor at reporting, or under‐report the severity of, their own symptoms of depression. Although the statistical methods used to test parent and child agreement on MFQ items in the ADHD sample suggested agreement ranged from slight to moderate, measuring agreement in this way is complex due to the differing cut offs used for parent and child report. As discussed, parents are expected to report fewer symptoms than their children, so perfect agreement is not expected. The especially interesting finding here is that children report fewer depression symptoms than their parents in the ADHD sample. This finding is important, as it may question the utility of relying only on child‐report questionnaires for depression amongst children and adolescents with ADHD.

It is possible that parents of children with ADHD over‐report or are more sensitive to depression symptoms in their children compared to parents of those without ADHD. Previous research has found that family functioning and child physical health are associated with a higher parent‐rated score of child emotional and behavioural problems (Collishaw et al., 2009), both of which could be factors that apply to this sample. However, previous literature examining self‐report of psychopathology in ADHD suggests that the difference may equally be coming from the child informant. Danckaerts et al. (1999) found that individuals with ADHD under‐report their own ADHD symptoms, and when Kooij et al. (2008) looked at the use of self‐report measures amongst adults with ADHD they found self‐report to be accurate, but sufferers tend to under‐report the severity of their own symptoms when compared to investigator‐rated clinical judgement. Children with ADHD have also been found to over‐estimate their competence in certain domains (Hoza, Pelham, Dobbs, Owens, & Pillow, 2002). The possibility that children with ADHD under‐estimate the severity of their conditions should therefore be taken into account when using depression screening tools in young people with ADHD.

When examining the level of agreement for each individual question in the ADHD group, the pattern showed that the lowest agreement came from the symptoms of restlessness, difficulty concentrating and feeling grumpy, while the highest agreement came from the symptoms of suicidal thoughts, and feelings that life was not worth living. Previous research has found that the former symptoms are amongst the strongest discriminating factors for depression, along with the core symptoms of depressed mood and anhedonia, while the latter symptoms are particularly important in identifying high severity depression (Cole et al., 2011). This indicates that while caution is needed when relying solely upon child‐report information to identify depression in ADHD, the fact that agreement is higher for these high risk symptoms may allow us to place more confidence in using either or both informants to identify high severity depression.

### Limitations

Firstly, there is a large over‐representation of boys in the ADHD sample. While this is representative of an ADHD population (as ADHD is more prevalent in males), affective disorders are more common in females (Wade, Cairney, & Pevalin, 2002) and can manifest differently depending on gender. However, when analyses were done separately by gender, the same pattern of results was found‐ both in terms of differences in MFQ scores between samples, and in differences in parent and child reporting of depression symptoms.

Secondly, the age distributions of the two groups are not closely matched, with the ADHD group having a higher mean age than the comparison group. Depression becomes more common as a child progresses into adolescence (Merikangas, Nakamura, & Kessler, 2009), therefore a sample with more similar age distributions may be more helpful for direct comparison. However, despite this, the differences between the total MFQ scores in our two samples remained even after the oldest participants from the ADHD sample were removed.

Finally, another important limitation to note is that the MFQ is a screening tool for depression symptoms, not a method used to diagnose depression. Therefore, although the MFQ scores in this study suggest depression symptoms are more common in an ADHD sample than in the general population, we cannot from these data conclude that depression diagnosis is more common.

## Conclusions

These findings provide evidence for the high co‐occurrence of depression symptoms with ADHD. Furthermore, they indicate that the symptoms of depression most commonly seen in ADHD are similar to the characteristic profile of a general population sample. Findings also suggest that children with ADHD may under‐report their depression symptoms when compared to those in a general population sample. This should be taken into account when screening children with ADHD for depression in a clinical setting.

## Ethical information

Ethical approval was obtained for the SAGE study from the Wales Multicentre Research Ethics Committee (MREC). Written informed consent from parents and assent from children (or consent if aged 16 and older) were obtained for all individuals. The twin study was approved by Wales MREC and Northwest MREC and consent was obtained from all participants following completion of the questionnaire.
